# Annotations

**Published:** 1910-07-02

**Authors:** 


### ANNOTATIONS.

### Women Students at General Hospital Schools.

THE current number of the Magazine of the London (Royal Free Hospital) School of Medicine for Women contains a short editorial on the subject of the suggested scheme, mooted in the "London Hospital Gazette," of opening the London to women students. The organ of the first women's medical school in the kingdom deprecates strongly the adoption of such a suggestion and points out that there are no valid arguments in its favour: "The large majority of students of the London (Royal Free Hospital) School of Medicine for Women," it remarks, "would be most strongly opposed to any change that would inevitably damage the school. Our own school and hospital provide us with more clinical material than we can fully avail ourselves of during our student course; and for those women who have already qualified, most of the special hospitals are available for post-graduate study. We fail to see that any further widening of the students' experience is either necessary or desirable. We do not wish to condemn the practice of men and women medical students working together, but we consider it more desirable that women should be trained apart from the men." This is a fair attitude to take up, although personal interest to some extent has entered into its consideration. The main advantage of opening all the medical schools in London must lie in the signal proof such opening would give of the fact that the profession no longer recognises the old false and crepuscular differentiation between the sexes so far as scientific study is concerned. For that reason we would welcome the cancellation of all existing restrictions which prevent women from entering as students at any medical school. Such cancellation, we are convinced, would not in the least degree interfere with the efficiency of the Royal Free School, whose reputation is such that no bid for popularity by any other London school where the majority of students must be men is likely to induce women to withdraw their support from it.

### Artesian Wells in the City.

IT has been well known for some time that various firms in the City of London have been making attempts to avoid the rather high charges of the Metropolitan Water Board by installing artesian wells of their own. The Medical Officer of Health for the area in question deals with this subject in his monthly report, adding some comments on the medical aspect involved. He states as an instance that the proprietors of a large West-End hotel, the water-rate of which was £900 a year, sank a well at a cost of £800, and found the annual cost of running the plant, including all repairs, to be only £200. Manifestly to firms able to pay the high rental of premises in London, a capital expenditure so moderate and effecting such a great saving, would be a mere fleabite, so that it is rather surprising to learn that in the City there are only 36 of these wells in use. However, as the report points out, if the supply proved insufficient the usual water-supply would have to be re-connected, and the cost of the operations lost. The quality of the water is stated to be excellent, as it is quite pure and much softer than that obtainable from public mains. The Medical Officer of Health considers that the advisability or otherwise of extending this practice is a matter rather outside his province, calling for the opinion of hydraulic engineers and geologists. We believe that artesian wells have proved of great service in the Colonies, about the only drawback, apart from the cost, being that in some cases the water obtained has a high temperature. Possibly experience might reveal other disadvantages in a very densely populated district.

### The Jubilee of Cinchona Culture in India.

IN the beginning of February 1860 cinchona-seeds were first brought from South America and sown in Southern India. The public benefactor who was instrumental in this was Sir Clements R. Markham, who solved the problem of acclimatisation by going personally to Peru and Bolivia to select cinchona plants and seeds, "enduring hornet stings and braving jaguars and snakes." So long as quinine salts were derivable almost entirely from cinchona bark produced in the plantations of the western hemisphere, prices ruled at figures which drained the pockets of the poor, inconvenienced controllers of medical institutions, and induced adulteration of a most pernicious nature. Frequently during the sixties the price of sulphate of quinine bordered on 20s. an ounce, and the purchasing public had to pay at a far higher rate for small quantities. But the enterprise of Sir Clements R. Markham and his helpers has subsequently so reduced the market price of this article that cinchona bark has frequently been at less than a fortieth of its price of fifty years ago. In his recent report on the trade and commerce of Java for 1909, Mr. Consul J. W. Stewart states, for example, that the average prices of sulphate of quinine sold at Batavia during 1907, 1908, and 1909 were respectively $6\frac{4}{5}$d., $6\frac{2}{5}$d., and $5\frac{3}{4}$d. per ounce.

### A Physician as Concierge.

ACCORDING to the *Centre Médical*, Louis XI. of France could think of no better way of rewarding his physician, Jacques Coitier, for the various services which the latter had rendered him than by conferring the title of "Comte des Cierges et de Bailli" upon the worthy medico. It must not, however, be imagined that the post was in any way derogatory to the dignity of a king's physician, for it is well known that when Hugues Capet decided to take up his abode at his city palace he caused it to be flanked by two buildings of considerable proportions, one of which was to contain the stables and coach-houses of the king under the direction of the Constable (*Comes Stabuli*), and the other, among other things, to contain the material necessary for the illumination of the palace, under the care of a noble captain who took the name of Comte des Cierges—that is to say, Concierge.

